# Case Report: Obsessive compulsive disorder in posterior cerebellar infarction - illustrating clinical and functional connectivity modulation using MRI-informed transcranial magnetic stimulation

**DOI:** 10.12688/wellcomeopenres.16183.2

**Published:** 2020-09-16

**Authors:** Urvakhsh Meherwan Mehta, Darshan Shadakshari, Pulaparambil Vani, Shalini S Naik, V Kiran Raj, Reddy Rani Vangimalla, YC Janardhan Reddy, Jaya Sreevalsan-Nair, Rose Dawn Bharath

**Affiliations:** 1 Department of Psychiatry, National Institute of Mental Health and Neurosciences, India, Bangalore, Karnataka, 560029, India; 2 Department of Neuroimaging and Interventional Radiology, National Institute of Mental Health and Neurosciences, India, Bangalore, Karnataka, 560029, India; 3 Cognitive Neuroscience Centre, National Institute of Mental Health and Neurosciences, India, Bangalore, Karnataka, 560029, India; 4 Graphics Visualization Computing Lab and E-Health Research Centre, International Institute of Information and Technology, Bangalore, Karnataka, 560100, India

**Keywords:** Obsessive Compulsive Disorder, Cerebellar cognitive affective syndrome, Neuromodulation, Functional brain connectivity, Cerebellar infarct, Theta burst stimulation

## Abstract

**Objectives: **We describe atypical and resistant neuropsychiatric clinical manifestations in a young male with posterior cerebellar gliosis. We also attempt to test the mediating role of the cerebellum in the clinical presentation by manipulating the frontal-cerebellar network using MRI-informed transcranial magnetic stimulation (TMS).

**Methods: **A case report of a young adult male describing obsessive-compulsive symptoms, probably secondary to an infarct in the cerebellar right crus II, combined with an examination of behavioral and functional connectivity changes following TMS treatment.

**Results: **Obsessions, compulsions, and pathological slowing were observed in the background of a posterior cerebellar infarct, along with impairments in vigilance, working memory, verbal fluency, visuospatial ability, and executive functions, in the absence of any motor coordination difficulties. These symptoms did not respond to escitalopram. MRI-informed intermittent theta-burst stimulation delivered to the pre-supplementary motor area identified based on its connectivity with the cerebellar lesion in the crus II resulted in partial improvement of symptoms with enhanced within and between-network modularity of the cerebellar network connectivity.

**Conclusion: **We illustrate a case of OCD possibly secondary to a posterior cerebellar infarct, supporting the role of the cerebellum in the pathophysiology of OCD. Functional connectivity informed non-invasive neuromodulation demonstrated partial treatment response. A seriation technique showed extended connectivity of the cerebellar lesion regions following the neuromodulatory treatment.

## Introduction

Cortico-striato-thalamocortical circuitry dysfunction is central to an integrated neuroscience formulation of obsessive-compulsive disorder (OCD) ^1, 2^. However, more recent large-scale brain connectivity analyses implicate the role of the cerebello-thalamocortical networks also ^3^. Here, we report a case of OCD secondary to a cerebellar lesion. We test the mediating role of the cerebellum in the manifestation of OCD by manipulating the frontal-cerebellar network using MRI-informed transcranial magnetic stimulation (TMS).

## Case report

A 21-year-old male, an undergraduate student from rural south India, presented to our emergency with suicidal thoughts. History revealed three years of academic decline, pathological slowness in routine activities (e.g., bathing, eating, dressing up, and using the toilet), repetitive ‘just-right’ behaviors (e.g., wiping his mouth after eating, clearing his throat, pulling down his shirt, mixing his food in the plate and walking back and forth until ‘feeling satisfied’). As a result, he spent up to three hours completing a meal or his toilet routines. Before presentation to us, he had received trials with two separate courses of electroconvulsive therapy (ECT) – six bitemporal ECTs at first, followed by nine bifrontal) spaced about two months apart. ECT was prescribed because of a further deterioration in his condition over the prior 18-months, with reduced oral intake, weight loss, grossly diminished speech output, and passing urine in bed (as he would remain in bed secondary to his obsessive ambitendency, as disclosed later). His oral intake and speech output improved with both ECT treatments, only to gradually worsen over the next few weeks. Given the potential catatonic phenomena (withdrawn behaviour and mutism) in the background of ongoing academic decline, slowness and stereotypies, he was also treated with oral olanzapine 20mg for eight weeks and risperidone 6mg for six weeks with minimal change in his slowness and repetitive behaviors. He did not receive any antidepressant medications. Psychotherapy was also not considered given the limited feasibility due to the severe withdrawal and near mutism. We could not elicit any contributory clinical history of prodromal or mood symptoms from adolescence when we evaluated his past psychiatric and medical history. Two months after the last ECT treatment, he presented to our emergency services with suicidal thoughts. He was admitted, and mental status examination revealed aggressive (urges to harm himself by jumping in front of a moving vehicle or touching electric outlets) and sexual obsessions with mental compulsions and passing urine in bed (as he could not go to the toilet in time due to obsessive ambitendency). The Yale-Brown Obsessive-Compulsive Scale (YBOCS) severity score was 29 ^4^. He had good insight into obsessions, but not the ‘just right’ repetitive behaviors; it was, therefore, challenging to engage him in psychotherapy. We treated him with escitalopram 40mg and brief psychoeducation before being discharged. After three months, his obsessions had resolved, but pathological slowness, ‘just right’ phenomena, and passing urine in bed had worsened (YBOCS score 31). 

**Figure 1.**
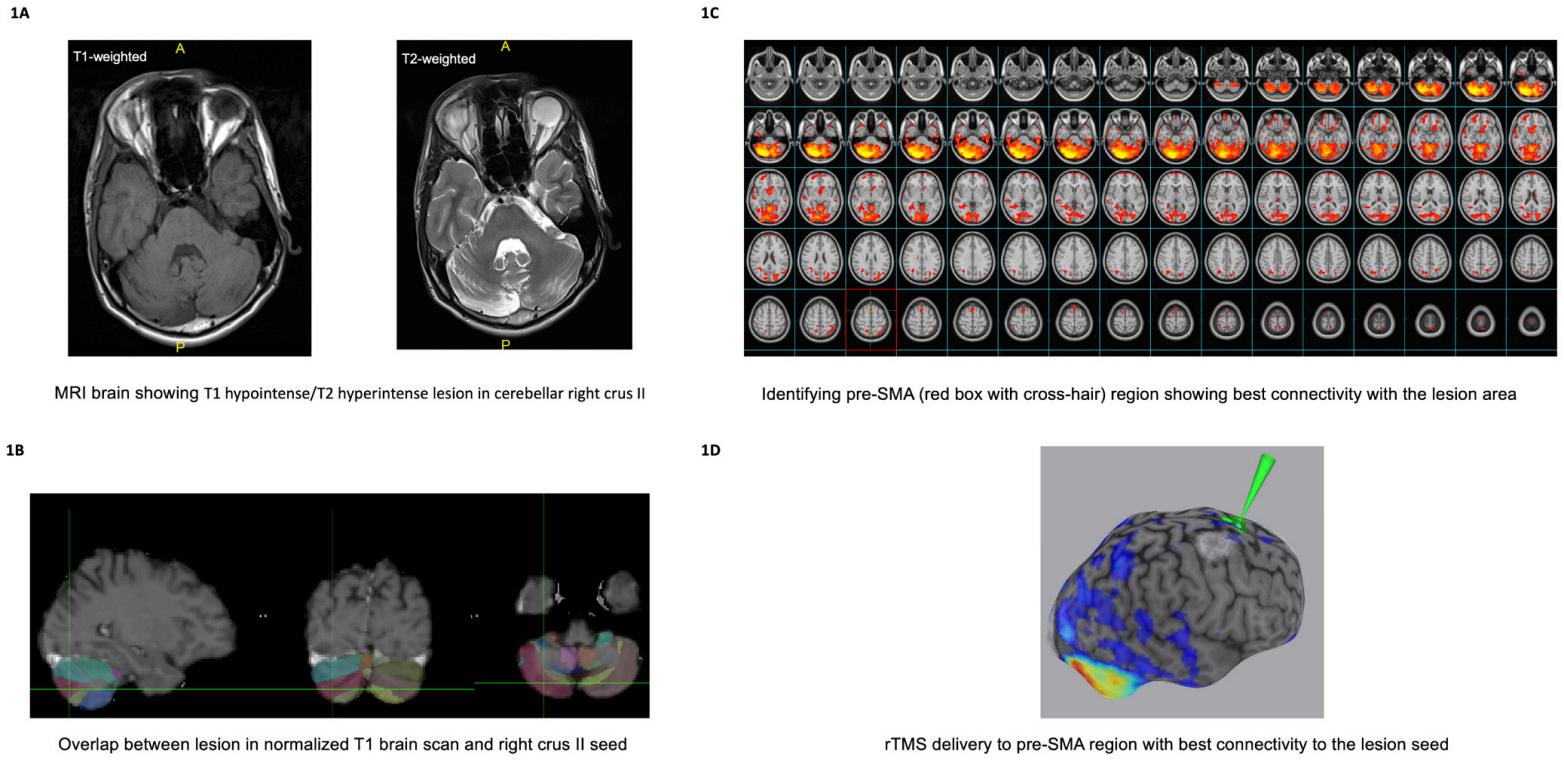
Cerebellar lesion detection ( **A** & **B**), its functional connectivity map ( **C**) and MRI-guided transcranial magnetic stimulation delivery ( **D**). Average blood oxygen level-dependent (BOLD) signal time-series were extracted from voxels within a binarized lesion-mask that overlapped with the right crus II ( ***1A*** & ***1B***). This was used as the model predictor in a general linear model to determine the brain regions that temporally correlated with the lesion-mask using FSL-FEAT ^11^. The resultant seed-to-voxel connectivity map (z-thresholded at 4) was used to identify the best connectivity of the seed with voxels in the pre-supplementary motor area (pre-SMA; MNI x=3; y=13; z=58; ***1C***). Six-hundred pulses were delivered as triplet bursts at theta frequency and 90% of the resting motor threshold (50 Hz; 2s on; 8s off) using a MagPro X100 (MagVenture, Denmark) device under MR-guided neuronavigation using the Brainsight stereotaxic system (Rogue Research, Montreal, Canada) with a figure-of-eight coil held with the handle in line with the sagittal plane, pointing toward the occiput to stimulate the pre-SMA site ( ***1D***).

We then obtained a plain and contrast brain MRI, to rule out an organic aetiology given the atypical nature of symptoms (apparent urinary incontinence) and the poor treatment response. The MRI revealed a wedge-shaped lesion in the right posterior cerebellum, suggestive of a chronic infarct in the posterior inferior cerebellar artery territory ( Figure-1A). MR-angiogram revealed no focal narrowing of intracranial and extracranial vessels. Electroencephalography, cerebrospinal fluid analysis, autoimmune and vasculitis investigations were unremarkable. Echocardiogram was normal and the sickling test for sickle cell anemia was also negative. We specifically inquired about history of loss of consciousness, seizures or motor incoordination, but these were absent. His neurological examination with a detailed focus on cerebellar signs was unremarkable. The International Cooperative Ataxia Rating Scale (ICARS) score was zero. The Cerebellar Cognitive Affective Syndrome (CCAS) scale revealed >3 failed tests – in domains of attention, category switching, response inhibition, verbal fluency, and visuospatial drawing, suggestive of definite CCAS ^5^.

## MRI-informed neuromodulation

Owing to inadequate treatment response and the possibility of OCD secondary to the cerebellar lesion, we discussed with the patient about MRI-informed repetitive transcranial magnetic stimulation (rTMS) and obtained his consent. The presence of a lesion involving a node (cerebellum) within the cerebello-thalamo-cortical circuit – a key pathway for error monitoring ^6^ and inhibitory control ^7^ – cognitive processes typically impacted in OCD prompted us to utilize a personalized-medicine approach to treatment. We acquired a resting-state functional-MRI echoplanar sequence (8m 20s; 250-volumes) in duplicate – before, and one-month after rTMS treatment on a 3-Tesla scanner (Skyra, Siemens), using a 20-channel coil with the following parameters: TR/TE/FA= 2000ms/30ms/78; voxel=3mm isotropic; FOV=192*192.

Image processing was performed using the FMRIB Software Library (FSL version-5.0.10) ^8^. Figure 1 describes how we obtained a seed-to-voxel connectivity map to identify the best connectivity of the cerebellar lesion-seed with voxels in the pre-supplementary motor area (pre-SMA; MNI x=3; y=13; z=58) – a commonly used site for neuromodulation in OCD ^9^. This area demonstrates connections with the non-motor (ventral dentate nucleus) parts of the posterolateral cerebellum ^10^ and contributes to error processing and inhibitory control along with the cerebellum ^7^. 

We augmented escitalopram with rTMS, administered as intermittent theta-burst stimulation (iTBS) to the pre-SMA coordinates ( Figure-1D). Six-hundred pulses were delivered as triplet bursts at theta frequency and 90% of the resting motor threshold (50 Hz; 2s on; 8s off) using a MagPro X100 (MagVenture, Farum, Denmark) device under MR-guided neuronavigation using the BrainSight stereotaxic system (Rogue Research, Montreal, Canada) with a figure-of-eight (MagVenture MCF-B-70) coil held with the handle in line with the sagittal plane, pointing toward the occiput to stimulate the pre-SMA site. We hypothesized that iTBS ^12^ to the pre-SMA could adaptively engage the cerebellum lesion, with which it shares neuronal oscillation frequencies, and hence improve the disabling symptoms. He received 27 iTBS sessions, once daily over the next month. Following ten sessions, he began to show a reduction in his repetitive behaviors, and by the 15 ^th^ session, he acknowledged that his behaviors were irrational. The YBOCS severity score had reduced to 24 (~22.5% improvement), which remained the same, even at the end of 27 sessions of iTBS treatment. There was no change in the CCAS and ICARS scores. The clinical benefits remained unchanged until three months of follow-up. Subsequently, we observed a gradual reversal to pre-TMS symptom severity. Maintenance TMS was suggested but was not feasible due to logistic reasons and therefore he was initiated on oral fluoxetine that was gradually increased to 80mg/day, with which we observed minimal change in symptoms over the next four months.

## Post-neuromodulation functional connectivity visualization

The pre- and post-rTMS scans ^13^ were parcellated into 48-cortical, 15-subcortical, and 28-cerebellar regions as per the Harvard-Oxford ^14^ and the Cerebellum MNI-FLIRT atlases ^15^. Average BOLD-signal time-series from each of these nodes, obtained after processing within FSL version-5.0.10, were then concatenated to obtain a Pearson’s correlation matrix between 91 nodes, separately for the pre- and post-TMS studies.

**Figure 2.**
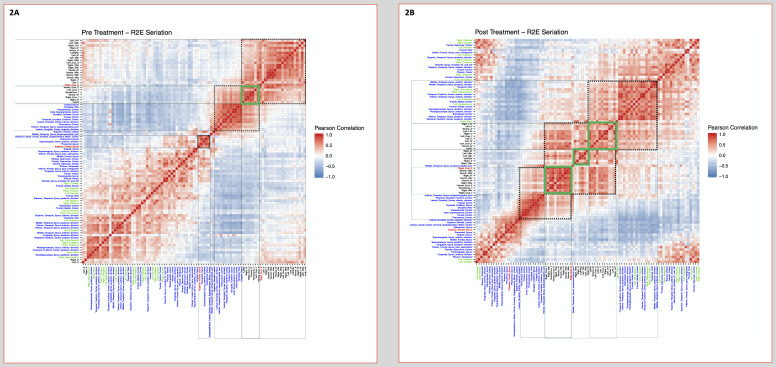
Rank-two ellipse seriation-based visualization of correlation matrix before ( **A**) and after ( **B**) rTMS treatment. The dotted-black boxes denote the cerebellar network and other connected networks, where the green boxes show the inter-network overlap. Thus, we see that the overlapped region in ( **2A**) has now transitioned to three different overlapped areas in ( **2B**), which shows the increase in the overlap between modular networks after treatment. Cerebellar nodes are denoted in black, cortical nodes in blue and subcortical nodes in green. The lesion node (right crus II) and the region of neuro-stimulation are given in red; R2E= Rank-two ellipse.

We analyzed the two 91 × 91 matrices using the Rank-two ellipse (R2E) seriation technique for node clustering ^16^ ( Figure 2). This technique reorders the nodes by moving the ones with a higher correlation closer to the diagonal. Thus, blocks along the diagonal of the matrix visualization show possible functional coactivating clusters.

We observed (a) extended connectivity of the cerebellar network after iTBS treatment as evidenced through its diminished modularity – the larger cerebellar cluster/block had an increased overlap with both anterior and posterior brain networks as observed along the diagonal in ( Figure 2B), and (b) formation of better-defined sub-clusters within the larger cerebellar cluster indicating improved within-network modularity of distinct functional cerebellar networks [e.g., vestibular (lobules IX and X) and cognitive-limbic (crus I/II and vermis)].

## Conclusions

We illustrate a case of OCD possibly secondary to a posterior cerebellar infarct, supporting the role of the cerebellum in the pathophysiology of OCD ^3^. That OCD was perhaps secondary to the posterior cerebellar lesion is supported by several lines of evidence. Firstly, there seemed to be a possible temporal correlation between the duration of OCD and the chronic nature of the cerebellar lesion. Despite the challenges in inferring a precise temporal relationship based on clinical history, the signal changes with free diffusion and atrophy indicated that the infarct was indeed chronic, supporting the symptom onset at about three years before presentation. Previous studies have indeed reported OCD in posterior cerebellar lesions ^17– 19^. Secondly, the clinical phenotype was somewhat atypical, characterized by severe ambitendency, precipitating urinary incontinence, and poor insight into compulsions along with comorbid CCAS. Thirdly, our patient was resistant to an anti-obsessional medication but improved partially with neuromodulation of the related circuit. The MRI-informed iTBS engaged the lesion-area by targeting its more superficial connections in the frontal lobe. The changes in clinical observations paralleled the changes in cerebellar functional connectivity – enhanced within-cerebellum modularity and expanded cerebellum to whole-brain connectivity.

This report adds to the growing evidence-base for the involvement of the posterior cerebellum in the pathogenesis of OCD. Drawing conclusions from a single case study and the absence of a placebo treatment will prevent any confirmatory causal inferences from being made. The opportunity to examine network-changes that parallel therapeutic response in an individual with lesion-triggered psychiatric manifestations not only helps mapping symptoms to brain networks at an individual level ^13^ but also takes us a step further to refine methods to deliver more effective personalized-medicine in the years to come. 

## Data availability

### Underlying data

Harvard Dataverse: PICA OCD Raw fMRI files NII format. https://doi.org/10.7910/DVN/X12BZD ^20^.

This project contains the following underlying data:

- postTMS_fmri.nii (raw post TMS fMRI file)
- preTMS_fmri.nii (Raw pre TMS fMRI file)

### Reporting guidelines

Harvard Dataverse: PICA OCD case report CARE guidelines for case reports: 13-item checklist. https://doi.org/10.7910/DVN/2XKSXL ^21^.

Data are available under the terms of the Creative Commons Zero "No rights reserved" data waiver (CC0 1.0 Public domain dedication).

## Consent

Written informed consent for publication of their clinical details and clinical images was obtained from the patient.
