# Supplementary material for: Case Report: Obsessive compulsive disorder in posterior cerebellar infarction - illustrating clinical and functional connectivity modulation using MRI-informed transcranial magnetic stimulation
Source: Wellcome Open Res. 2020 Sep 16;5:189. Originally published 2020 Aug 10. [Version 2] doi: 10.12688/wellcomeopenres.16183.2 (PMC7503177; doi:10.12688/wellcomeopenres.16183.2)
Supplement: Peer review history

# Peer review: Reviewer response for version 2

**DOI:** 10.21956/wellcomeopenres.17923.r40485

**Published:** 2020-09-18

**Authors:** Peter Enticott

**Affiliations:** 1 School of Psychology, Deakin University, Geelong, Vic, Australia

Thank you for your considered response to my review; I have no further comments.

Are enough details provided of any physical examination and diagnostic tests, treatment given and outcomes?

Yes

Is the case presented with sufficient detail to be useful for other practitioners?

Yes

Is sufficient discussion included of the importance of the findings and their relevance to future understanding of disease processes, diagnosis or treatment?

Partly

Is the background of the case’s history and progression described in sufficient detail?

Partly

Reviewer Expertise:

Cognitive neuroscience

I confirm that I have read this submission and believe that I have an appropriate level of expertise to confirm that it is of an acceptable scientific standard.

# Peer review: Reviewer response for version 1

**DOI:** 10.21956/wellcomeopenres.17767.r39915

**Published:** 2020-09-04

**Authors:** Peter Enticott

**Affiliations:** 1 School of Psychology, Deakin University, Geelong, Vic, Australia

This is a very interesting case report, even without the intervention component (which itself is a fascinating approach to neuromodulation). I particularly appreciated the approach to regional (SMA) targeting, which involved resting state fMRI to detect functional connectivity with the affected cerebellar region. The report itself is very clear and well-written.

 ECT appears to have been provided in the context of a depressive episode, but were other (e.g., psychotherapy, pharmacotherapy) treatments initially trialled? It would be useful to present any clinical history from adolescence, although this may not be feasible.

 Please describe the reason for conducting MRI; why was this not undertaken earlier?

 Was iTBS the “standard” course (i.e., 600 pulses, trains comprising 3 pulses at 50 Hz, repeated for 2 seconds at 5 Hz, followed by an 8-second ITI)? How was intensity determined (e.g., 70%RMT, 80%AMT)? Specify the stimulator, coil type, and neuronavigation method.

 Given that the duration of both the cerebellar lesion and OCD symptoms seems quite unclear, it is somewhat difficult to suggest a temporal relationship (as stated in the Conclusion).

 Was the patient followed-up over a longer-term period? I would be interested to know if these improvements are lasting (i.e., longer than 3 months), although again this might not be possible.

Are enough details provided of any physical examination and diagnostic tests, treatment given and outcomes?

Yes

Is the case presented with sufficient detail to be useful for other practitioners?

Yes

Is sufficient discussion included of the importance of the findings and their relevance to future understanding of disease processes, diagnosis or treatment?

Partly

Is the background of the case’s history and progression described in sufficient detail?

Partly

Reviewer Expertise:

Cognitive neuroscience

I confirm that I have read this submission and believe that I have an appropriate level of expertise to confirm that it is of an acceptable scientific standard, however I have significant reservations, as outlined above.

# Response

**Published:** 2020-09-08

**Authors:** Urvakhsh Mehta

**Affiliations:** National Institute of Mental Health and Neurosciences, India, India

- This is a very interesting case report, even without the intervention component (which itself is a fascinating approach to neuromodulation). I particularly appreciated the approach to regional (SMA) targeting, which involved resting state fMRI to detect functional connectivity with the affected cerebellar region. The report itself is very clear and well-written.

>>>> We thank the reviewer for the time taken to provide constructive feedback and the encouraging comments on this report. 

  

- ECT appears to have been provided in the context of a depressive episode, but were other (e.g., psychotherapy, pharmacotherapy) treatments initially trialled? It would be useful to present any clinical history from adolescence, although this may not be feasible.

>>>> Thank you for this important clarification. As mentioned in the manuscript, ECT was prescribed by the earlier treating psychiatrist because of a further deterioration in his condition over the prior 18-months, with reduced oral intake, weight loss, grossly diminished speech output, and passing urine in bed (as he would remain in bed secondary to his obsessive ambitendency). Given the potential catatonic phenomena (withdrawn behaviour and mutism) in the background of ongoing academic decline, slowness and stereotypies, he was also treated with oral olanzapine 20mg for eight weeks and risperidone 6mg for six weeks with minimal change in his slowness and repetitive behaviors before he presented to us. *He did not receive any antidepressant medications. Psychotherapy was also not considered given the limited feasibility due to the severe withdrawal and near mutism. We could not elicit any contributory clinical history of prodromal or mood symptoms from adolescence when we evaluated his past psychiatric and medical history. *We have now clarified this more elaborately in the first paragraph of the *case report* section. 

  

- Please describe the reason for conducting MRI; why was this not undertaken earlier?

>>>> MRI was conducted for two reasons – *poor treatment response and atypical symptoms (apparent urinary incontinence) *that could mimic an underlying neurological aetiology. We have explicitly stated this in the revised manuscript now in the second paragraph of the *case report* section. 

  

- Was iTBS the “standard” course (i.e., 600 pulses, trains comprising 3 pulses at 50 Hz, repeated for 2 seconds at 5 Hz, followed by an 8-second ITI)? How was intensity determined (e.g., 70%RMT, 80%AMT)? Specify the stimulator, coil type, and neuronavigation method.

>>>> The iTBS details were provided in the figure-1 legend as follows: *Six-hundred pulses were delivered as triplet bursts at theta frequency and 90% of the resting motor threshold (50 Hz; 2s on; 8s off) using a MagPro X100 (MagVenture, Farum, Denmark) device under MR-guided neuronavigation using the BrainSight stereotaxic system (Rogue Research, Montreal, Canada) with a figure-of-eight (MagVenture MCF-B-70) coil held with the handle in line with the sagittal plane, pointing toward the occiput to stimulate the pre-SMA site. *These have now been stated in the third paragraph of the *MRI-informed neuromodulation* section in the revised manuscript. 

  

- Given that the duration of both the cerebellar lesion and OCD symptoms seems quite unclear, it is somewhat difficult to suggest a temporal relationship (as stated in the Conclusion).

>>>> We do concede that the temporal relationship appears tenuous. However, the signal changes with free diffusion and atrophy indicated it was a chronic lesion, supporting the symptom onset at about three years before presentation. In this context, we provide these additional objective MRI details in the revised manuscript and acknowledge the challenges in inferring a precise temporal relationship based on clinical history. The revised text in the *conclusion* section appears as follows:

*“Firstly, there seemed to be a possible temporal correlation between the duration of OCD and the chronic nature of the cerebellar lesion. Despite the challenges in inferring a precise temporal relationship based on clinical history, the signal changes with free diffusion and atrophy indicated that the infarct was indeed chronic, supporting the symptom onset at about three years before presentation.”*

  

- Was the patient followed-up over a longer-term period? I would be interested to know if these improvements are lasting (i.e., longer than 3 months), although again this might not be possible. 

>>>> Since the time the report was initially drafted, we have further followed up the patient. *The clinical benefits remained unchanged until three months of follow-up. Subsequently, we observed a gradual reversal to pre-TMS symptom severity. Maintenance TMS was suggested but was not feasible due to logistic reasons and therefore he was initiated on oral fluoxetine that was gradually increased to 80mg/day, with which we observed minimal change in symptoms over the next four months.* These details have now been provided in the last paragraph of the *MRI-informed neuromodulation* section in the revised manuscript. 

# Peer review: Reviewer response for version 1

**DOI:** 10.21956/wellcomeopenres.17767.r40234

**Published:** 2020-09-02

**Authors:** Shubhmohan Singh

**Affiliations:** 1 Department of Psychiatry, Postgraduate Institute of Medical Education and Research, Chandigarh, Punjab and Haryana, India

This is an interesting case report that deals with a novel method of neuronavigation based rtms. 

 Within the limitations imposed by the accidental nature of the lesion discovery and the tenuous nature of speculation on causation and correlates of the modest improvement, this is a good demonstration of this particular technique.

Are enough details provided of any physical examination and diagnostic tests, treatment given and outcomes?

Yes

Is the case presented with sufficient detail to be useful for other practitioners?

Yes

Is sufficient discussion included of the importance of the findings and their relevance to future understanding of disease processes, diagnosis or treatment?

Yes

Is the background of the case’s history and progression described in sufficient detail?

Yes

Reviewer Expertise:

Neuromodulation, psychiatry

I confirm that I have read this submission and believe that I have an appropriate level of expertise to confirm that it is of an acceptable scientific standard.

# Response

**Published:** 2020-09-08

**Authors:** Urvakhsh Mehta

**Affiliations:** National Institute of Mental Health and Neurosciences, India, India

We thank this reviewer for the time taken to provide constructive feedback and the encouraging comments on this report.
